# Unusual bilateral ovarian metastases from ileal gastrointestinal stromal tumor (GIST): a case report

**DOI:** 10.1186/s12885-018-4204-1

**Published:** 2018-03-16

**Authors:** Antonio De Leo, Margherita Nannini, Giulia Dondi, Donatella Santini, Milena Urbini, Elisa Gruppioni, Pierandrea De Iaco, Anna Myriam Perrone, Maria Abbondanza Pantaleo

**Affiliations:** 1 Pathology Unit, S.Orsola-Malpighi Hospital, Via Massarenti 9, 40138 Bologna, Italy; 2 Department of Specialized, Experimental and Diagnostic Medicine, S.Orsola-Malpighi Hospital, University of Bologna, Via Massarenti 9, 40138 Bologna, Italy; 3 Gynecologic Oncology Unit, S.Orsola-Malpighi Hospital, Via Massarenti 9, 40138 Bologna, Italy; 4 “Giorgio Prodi” Cancer Research Center, University of Bologna, Bologna, Italy; 5 Laboratory of Oncologic Molecular Pathology, S.Orsola-Malpighi Hospital, Via Massarenti 9, 40138 Bologna, Italy

**Keywords:** GIST, Ovarian metastases, Hysterectomy, Bilateral annexiectomy

## Abstract

**Background:**

Gastrointestinal stromal tumors (GIST) are the most common mesenchymal tumors of the gastrointestinal tract and liver and peritoneum are the main sites of recurrence. Ovarian metastases from GIST are very rare.

**Case Presentation:**

A 50 years-old woman was found to have a pelvic mass on transvaginal ultrasound (TV-US) and computed tomography (CT)-scan, considered as a right ovarian mass. The patient underwent surgical abdominal exploration that showed an ileal mass, a normal right ovary and an irregular and vascularized surface of the left ovary. A segmental ileal resection and an ileal anastomosis were performed. Frozen section showed a GIST and surgery was completed with hysterectomy, bilateral salpingo-oophorectomy, pelvic peritonectomy, peritoneal washing and Burch procedure. The histological examination confirmed an ileal GIST with ovarian metastases, harboring in both sites of disease a KIT exon 11 deletion.

**Conclusions:**

Ovarian localizations, as far as rare, can be a clinical finding in case of ileal GIST patients, and both gynecologists, pathologists and medical oncologists should be able to recognize them.

**Electronic supplementary material:**

The online version of this article (10.1186/s12885-018-4204-1) contains supplementary material, which is available to authorized users.

## Background

Gastrointestinal stromal tumors (GIST) are the most frequent mesenchymal tumors of the gastrointestinal tract, commonly occurring in the stomach (60%), followed by the small bowel (35%) and other sites (colon, rectum, and esophagus < 5%) [1]. Nevertheless, they are considered rare, with an incidence in Europe of 1.5 new cases per million per year.

As is well known, the liver and peritoneum are the most frequent site of metastases, both as synchronous and metachronous presentation [2]. Even if more rarely, also lung and bone metastases can occur during the disease course [3, 4]. In the past years, other anecdotal sites of GIST recurrences have been reported in single cases [5–8]. To our knowledge, ovarian metastases from GIST have been previously described only in five cases in a series [9].

Herein we report a case of GIST patient presenting bilateral ovarian metastases at the onset of disease, highlighting the pathological/molecular features of this unusual site of metastatic presentation and the clinical implications.

## Case Presentation

**Fig 1:**
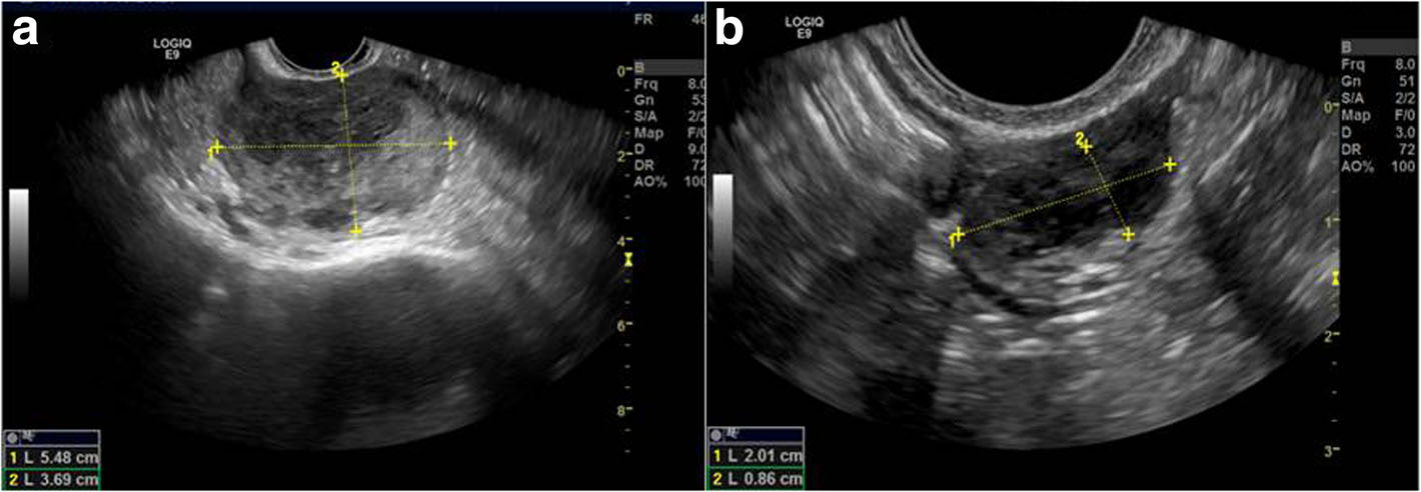
**a** The solid and hypoechoic pelvic mass detected at trans-vaginal ultrasound. **b** Ultrasonographic aspect of left ovary

**Fig. 2:**
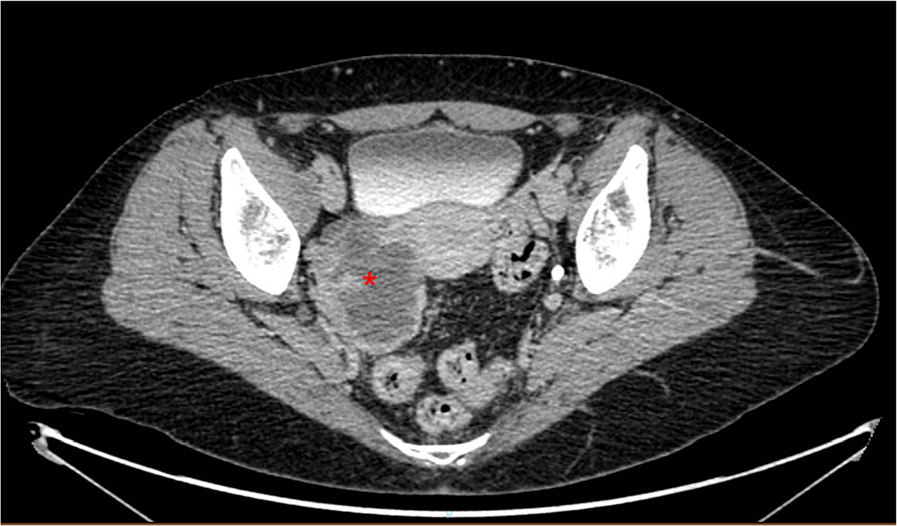
CT scan image. The single star shows the pelvic mass

**Fig 3:**
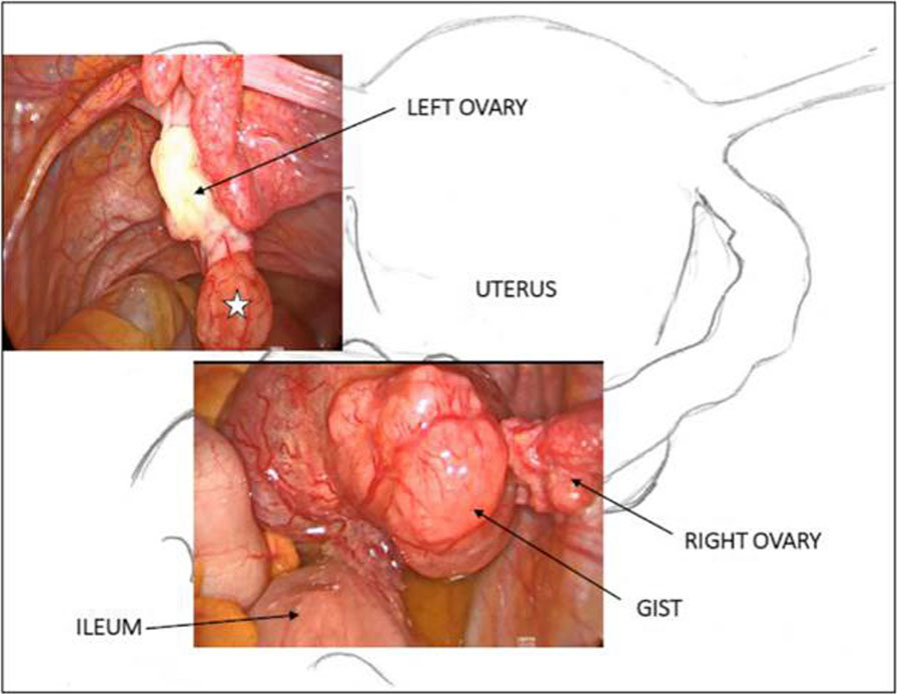
The laparoscopic pictures show the pelvic mass originating from the ileum and the exophytic vegetation (single star) of the left ovary that resulted a GIST metastasis at final microscopic examination

A 50 years-old woman, G1P1 was referred to gynecological consultation for uterine prolapse. Her medical history was positive for a spinal schwannoma, surgically removed the year before and her family history was negative for cancer. Transvaginal ultrasound (TV-US) showed a solid hypoechoic pelvic mass, with irregular margins, vascularized to power Doppler (color score 3), not fixed to surrounding structures (Fig. 1a). Because of the site and the proximity to the ovarian vessels it was considered as a right ovarian mass of 54 × 37 mm of diameter. Left ovary appeared normal except to two small anechoic images (< 1 mm) mimicking ovarian follicles (Fig. 1b). The subsequent computed tomography (CT)-scan evaluation confirmed the presence of an expansive right adnexal lesion, with lobulated margins and heterogeneous enhancement on post-contrast CT images (Fig. 2). The woman was counseled to perform hysterectomy and bilateral salpingo-oophorectomy with intraoperative frozen section on right adnexa and simultaneous correction of prolapse with laparoscopic access. Thus, the patient underwent surgery. Laparoscopic abdominal exploration showed a round shaped, reddish, easily bleeding mass that originated from one ileal loop displaced in the pelvis. Right ovary was normal and covered by the mass, left ovary presented a normal volume but an irregular and vascularized surface and a round shaped, reddish, exophytic vegetation of 1 cm of diameter originated from the ovarian surface. Small nodules with similar appearance of the pelvic mass suspected for diffusion of disease were showed in the peritoneum of the pouch of Douglas (Fig. 3a and b).

**Fig. 4:**
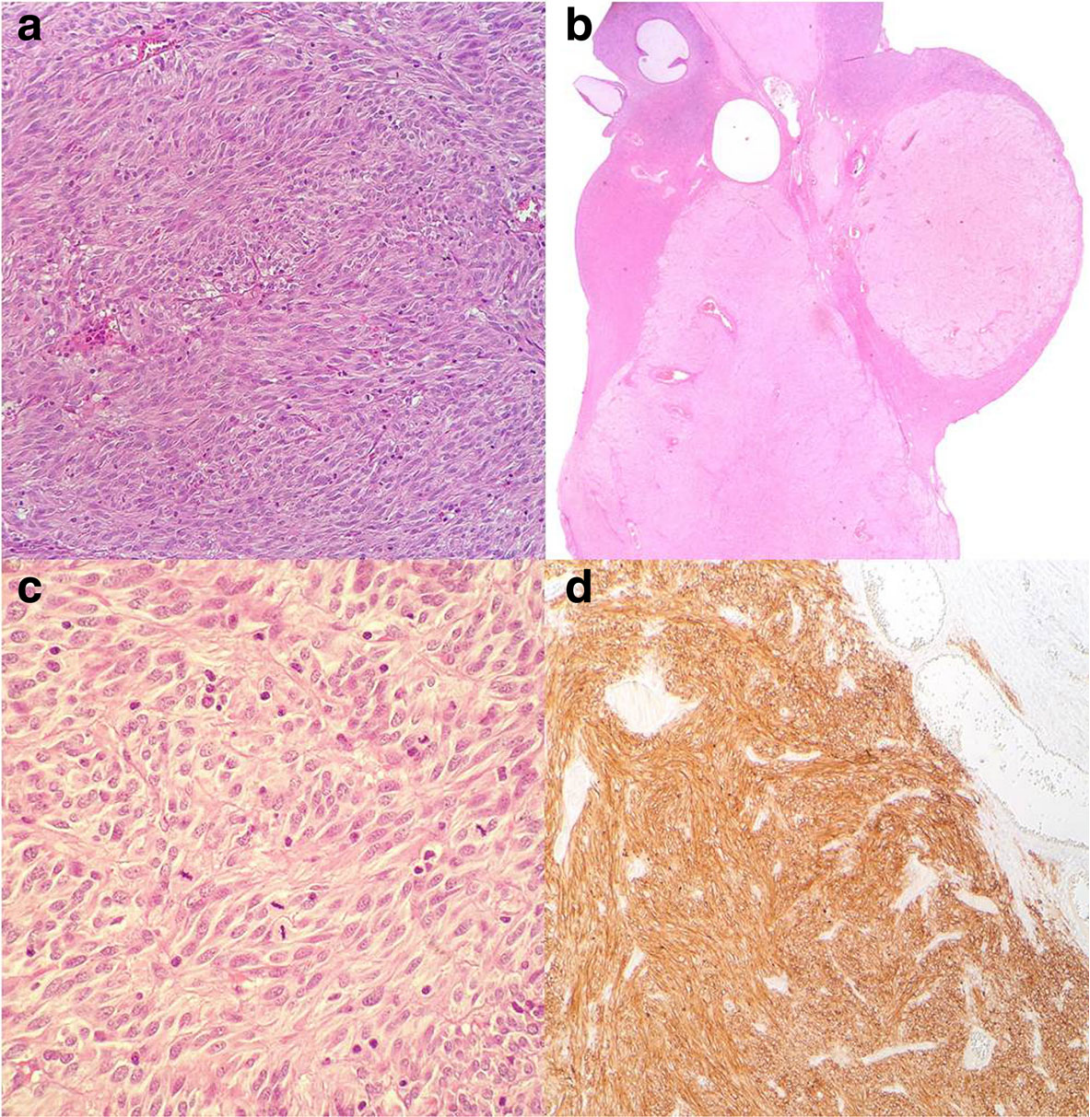
**a** Ileal GIST, Hematoxylin and Eosin (H&E) stain, original magnification × 200. **b** Left ovary, Hematoxylin and Eosin (H&E) stain, original magnification × 20. **c** Left ovary, Hematoxylin and Eosin (H&E) stain, original magnification × 400. **d** Left ovary, DOG-1, original magnification × 100

In consideration of the size and the uncertain nature of the mass, of the suspicion of peritoneal diffusion, the surgeon decided to carry out a laparotomy. The explorative laparotomy shows that others peritoneal surfaces and organs were free from disease and no enlarged retroperitoneal lymph nodes were detected. Patient was submitted to removal of the mass with ileal loop resection and ileal anastomosis without stoma. Frozen section showed a GIST. Surgery was completed with hysterectomy, bilateral salpingo-oophorectomy, pelvic peritonectomy, peritoneal washing and Burch procedure. At macroscopic examination, the pathological examination revealed an ileal tumor mass measuring 7 × 5 × 5 cm and both ovaries showed two bilateral nodular lesions of 2 cm and 0.5 cm, respectively. The ileal mass was solid, soft, pink-tan tissue with areas of hemorrhage. Microscopic examination of the small bowel tumor showed circumscribed proliferation of intersecting fascicles of spindle cells with moderate atypia. Mitoses numbered 19 per 50 *High Power Field* (HPF); occasional atypical mitotic figures were noted. Immunohistochemistry showed strong and diffuse positivity for DOG-1 and c-kit leading to the diagnosis of GIST. The ovarian nodules, grossly yellowish, solid, with circumscribed borders, were composed, on microscopy, by a proliferation of stromal spindle cells arranged in short fascicles, with pale, occasionally vacuolated eosinophilic cytoplasm and mild atypia. Considering the coexisting ileal GIST, immunohistochemical stains for DOG-1 and c-kit were also performed resulting strongly and diffusely positive. The uterus was unremarkable. Cytology from peritoneal washing was negative. The final diagnosis was GIST (spindle cell type) of small bowel with bilateral ovarian metastases (Fig. 4a, b, c, d) [10].

Molecular analysis performed on the primary tumor by direct Sanger sequencing revealed a KIT exon 11 c-1653_1670del mutation, with a consequent p.M552_W557 deletion. Then we performed the mutational analysis on all sites of disease, including the left ovary lesion, confirming the same KIT exon 11 deletion of the primary, without secondary mutations.

The patient was referred to the GIST Study Group of our Institution for an appropriate clinical management and follow-up. A daily treatment with imatinib at the standard dose of 400 mg was then started, with a surveillance program by CT-scan every 4 months. At the last follow-up the patient is still alive and free of disease (Additional file 1).

## Discussion and conclusions

The most common metastatic sites of GIST are the liver and the peritoneum [2]; more rarely bone and lung can occur [3, 4]. Ovarian metastases are exceptionally rare and have been previously described only in five cases [9].

Herein we reported a case of GIST patient, presenting ovarian metastases at the onset of disease. Given the rarity of this unusual presentation, we retrospectively reviewed the clinical cases of our institutional reference center for GIST and among 224 female patients collected from 2001, we found only another case of GIST patient with synchronous ovarian metastases who was managed at the time of diagnosis in another center. GIST primary site was the ileum in both cases and the ovarian localizations were multiple and of small dimension, ranging to 0,5 cm to 2 cm of diameter. Similarly, the ovarian nodules, grossly yellowish, solid, with circumscribed borders, were composed on microscopy by a proliferation of stromal spindle cells arranged in short fascicles, with pale, occasionally vacuolated eosinophilic cytoplasm and mild atypia (Fig. 4a, b, c, and d). A KIT exon 11 c.1674_1695del mutation with a consequent Lys558_Gly565 deletion was found by direct Sanger sequencing [10].

Taken together, in addition to the unusual clinical presentation, these cases may raise some cues of discussion that may be relevant in clinical practice. From a pathological point of view, gross examination showed different appearances of primary GIST and ovarian localization. The ileal mass had the common macroscopic features of a GIST, but the ovarian lesions exhibited yellowish color and circumscribed borders suggesting a fibroma/thecoma. Although the microscopic features were similar in ileal and ovarian lesions, considering the unusual pattern of spread, differential diagnostic considerations should be made. The diagnosis of benign or malignant spindle cell tumor, primary or metastatic from the uterus or other non-genital tract site, should be initially considered. The differential diagnosis included benign or malignant ovarian stromal tumors (e.g. fibroma, thecoma, fibrosarcoma), granulosa cell tumor with fibromatous stroma, localization of leiomyoma or metastasis of sarcoma (e.g. leiomyosarcoma, endometrial stromal sarcoma). In order to establish the correct diagnosis, accurate macroscopic and microscopic examination and appropriate immunohistochemistry are crucial.

Regarding the kinase genotype, both cases harbor a KIT exon 11 mutation, that represents the most common molecular event of GIST [11]. Of note, in both cases the kind of mutation is an exon deletion, that it is already known to correlate with a poorer clinical outcome, if compared to insertion or point mutation mutations, in line with the metastatic presentation of both cases [12].

From a radiological point of view, the routine gynecological exam by TV-US was at first apparently negative. Left ovary was described with normal features and some ovarian follicles and pelvic mass was misdiagnosed as right ovary. In the light of the pathological examination, US-images were thus revised and subtle differences between ovarian follicles and ovarian metastases from GIST were found. Both are round-shaped and have similar dimension (5–10 mm) but the first are totally anechoic, while the second are hypoechoic without abnormal vascularization at power doppler (color score 2). Compared to ovarian metastases from other primary cancers, accounting for about 16% of all ovarian malignancies, some radiological differences can be found. As is well known, ovarian metastases can arise from both non-gynecologic tumors, as colon cancer (30%), stomach (16%), appendix (13%), breast (13%), pancreas (12%) and biliary tract (15%) cancers, and gynecologic tumors as uterus (23%) and cervix (4%) cancers [13]. Ovarian metastases from gastric, breast and uterus cancers appear as solid masses at TV-US and a typical feature is the presence of a leading central vessel, while those from colorectal and biliary tract cancers are more heterogeneous and often they appear as multilocular solid masses [14]. Conversely, as far as we know, ovarian metastases from GIST tend to have different US features. In particular, GIST ovarian metastases seem to be multifocal and located on the ovarian surface, while metastases from other carcinomas are deeper. However, more data on US aspects of ovarian metastases from GIST should be collected, in order to better recognize them during both pre-operative work up and during follow up of GIST patients. The role of CT-PET scan in this setting should be explored, even if the small size of the lesions may reduce the sensitivity, specificity, and accuracy of this exam for detecting metastatic ovarian lesions from GIST [15].

In conclusion, ovarian localizations, as far as rare, can be a clinical finding in case of ileal GIST patients, and both gynecologists, pathologists and medical oncologists should be able to recognize them. Given the low accuracy of CT-scan for detecting small ovarian lesions, due to their cystic pattern, a TV-US may be considered as a pre-operative exam for all female patients affected by ileal GIST, in order to exclude synchronous metastases, and also during the follow up, especially in case of doubtful ovarian findings. Finally, in case of clinical and macroscopic suspect of metastasis in the ovaries and, of course, taking into account the age of the patient, a wider radical gynecological surgery may be considered.

## Additional file

Additional file 1: The patient’s clinical history organized as a timeline. (PDF 304 kb)

## Abbreviations

CT-scan: Computed tomography scan
GIST: Gastrointestinal stromal tumor
HPF: High Power Field
TV-US: Transvaginal ultrasound
