# Supplementary material for: Unusual bilateral ovarian metastases from ileal gastrointestinal stromal tumor (GIST): a case report
Source: BMC Cancer. 2018 Mar 16;18:301. doi: 10.1186/s12885-018-4204-1 (PMC5857138; doi:10.1186/s12885-018-4204-1)
Supplement: Supplementary file 1 — The patient’s clinical history organized as a timeline. (PDF 304 kb) [file 12885_2018_4204_MOESM1_ESM.pdf]

November 2016

Uterine prolapse

TV-US

solid hypoechoic pelvic mass

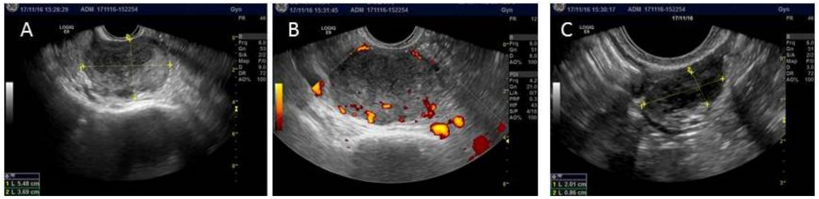

CT-scan

expansive right adnexal lesion, with lobulated margins and heterogeneous enhancement

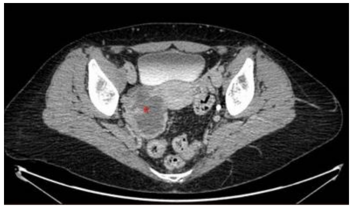

January 2017

Surgery

ileal loop resection, hysterectomy, bilateral salpingo-oophorectomy, pelvic peritonectomy, peritoneal washing

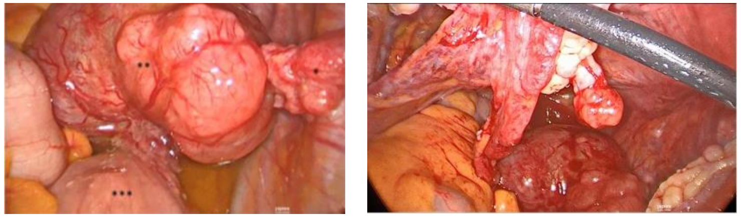

Diagnosis

GIST (spindle cell type) of small bowel with bilateral ovarian metastases

KIT exon 11 mutation (p.M552_W557)

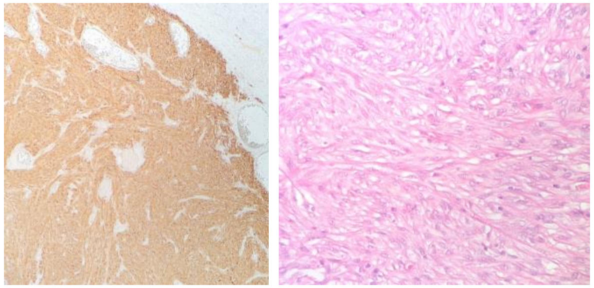

February 2017

Adjuvant imatinib

Alive, no recurrence
